# Factors Associated with Multi-Drug Resistant Tuberculosis among TB Patients in Selected Treatment Centers of Amhara Region: A Case-Control Study

**DOI:** 10.4314/ejhs.v31i1.4

**Published:** 2021-01

**Authors:** Getahun Yeshiwas Ambaye, Gebiyaw Wudie Tsegaye

**Affiliations:** 1 Department of Nursing, Dessie Health Science College, South Wollo, Ethiopia; 2 Department of Epidemiology and Biostatistics, Bahir Dar University, Bahir Dar, Ethiopia

**Keywords:** Ethiopia, multi drug resistant tuberculosis, determinants of microbial resistance

## Abstract

**Background:**

Multi-drug Resistant Tuberculosis (MDR-TB) is found to be a major public health problem both in developed and developing countries. Ethiopia is one of the 30 high MDR-TB burden countries in the world. Although several studies were done to identify the determinants of MDR-TB, the reported findings are heterogeneous across the world.

**Methods:**

Unmatched case-control study was conducted at Debre Markose Referral Hospital, Debre Birhan Referral Hospital, and Boru Media District Hospital in Amhara Region, Northern Ethiopia, from March 01/2019- April 30/2019. Cases were all tuberculosis patients with culture or line probe assay confirmed mycobacterium tuberculosis resistant to at least both Isoniazid and Rifampicin and registered on second-line TB treatment. Controls were all patients with Bacteriological (molecular) proven drug-susceptible TB strains and whose recent smears result were turned to negative and registered as cured from January 01/2014 – December 31/2018. A pre-tested checklist was used to collect the data.

**Result:**

Of the total reviewed documents (393), 98 cases and 295 controls were involved in this study. And, 54(55.1%) among cases and 162(54.9%) among controls were males. sixty nine(70.4%) among cases and 163(55.3) among controls were within the age group of 26–45 years. In the multivariable logistic regression analysis, age between 26–45 years old (AOR=3.35; 95% CI: 1.15, 9.77), previous history of TB treatment (AOR= 14.2; 95%CI: 7.8, 25.3) and being HIV positive (AOR=4.4; 95% CI: 1.8, 10.4) were significantly associated with MDR-TB.

**Conclusion:**

Age between 26–45 years old, previously treated cases and TB/HIV co-infection were identified as the determinants of MDR-TB. Special attention should be given to age between 26–45 years old, previous history of TB treatment and TB/HIV co-infection to prevent and control MDR-TB in the local context.

## Introduction

Tuberculosis (TB) is an infectious disease caused by Mycobacterium Tuberculosis complex which usually infects the lung. It is one of the top 10 causes of death worldwide and the leading cause of death from a single infectious agent (ranking above HIV/AIDS). It was responsible for 1.2 million deaths among HIV-negative and 251 000 deaths among HIV-infected people (1). Multi-Drug Resistant TB (MDR-TB) is defined as mycobacterium Tuberculosis resistant to at least Isoniazid and Rifampicin (2). Drug- resistant TB continues to be a public health threat both in developed and developing countries. In 2018, there were about half a million new cases of Rifampicin-Resistant TB of which 78% had MDR-TB (2,3). MDR-TB is progressively increasing among new and previously TB treated cases. Globally, 3.5% of new TB cases and 18% of previously treated cases had MDR/RR-TB (1). Although the overall prevalence of TB decreased by two percent, the incidence of MDR-TB remain increasing overtime (3,4). It is more common in resource limited regions due to poor adherence to treatment and lack of infection prevention practices both at home and in health facilities (5). Although 89.7% of the cases found in 30 high MDR-TB burden countries, it has been distributed in several regions in the world (2). According to WHO 2019 report, among all TB cases (10.million), 214,000 deaths were due to MDR-TB (1). A study conducted in South Africa in 2017 indicated that the mortality rate of MDR-TB patients was significantly high and more prevalent among previously TB treated cases (6). Ethiopia is among the 30 MDR-TB high burden countries with an estimated 5800 cases and prevalence rate of 21% among previously treated TB patients and 2.2% among newly diagnosed TB patients (2,7). On average, Ethiopia spent 75% health-related budget and 22.3% of annual household income on drug-resistant TB (8,9).

MDR-TB mainly affects uneducated, low socioeconomic status and reproductive age group of the population (13). Although TB is curable, patients infected with MDR strains are difficult to cure, and it remains a source of infection for a longer period (14). Poor adherence to TB treatment has been also observed in MDR-TB patients due to drug toxicity, high death rates (50–80%), serious psychological and economic crisis for infected individuals and their families (15). Several risk factors for MDR-TB were reported from different studies at different times in the world (16). Poor drug supply, use of inappropriate guidelines, poor treatment adherence, poor infection prevention practice, improper drug storage, wrong dose utilization, poor supervision by health professionals and close contact with MDR-TB cases were recognized as the determinants of MDR-TB (15–19).

Although some studies were conducted to identify the determinants of MDR-TB, the reported findings were heterogeneous in study designs and setting (15,18). Some variables like cigarette smoking (17, 20) and Human Immune Deficiency Virus (HIV) (20,22) have shown inconsistent relation with MDR-TB as reported from several studies in the past. For example, being HIV positive was listed as the risk factor for MDR-TB in some studies (21, 23, 24) while A systematic review and meta-analysis done on 32 studies reported no association between MDR-TB and HIV infection (22). Despite the fact that MDR-TB continued to be an emerging public health threat specially in areas with poverty, and high prevalence of HIV, there were limited studies in Amhara Region with smaller sample size and limited study setting (22.) Furthermore, the study area is characterized by high population growth, lifestyle change of population and immigration of a huge number of people from several regions of the country. These encourage the demand for an up-to-date large scale studies in the local context. Therefore, the purpose of this study was to identify the determinant of MDR-TB among TB patients at selected MDR-TB treatment initiating centers of Amhara Region, Northern Ethiopia.

## Methods and Materials

This unmatched case control study was conducted at three MDR-TB initiating centers (Debre Markose Referral Hospital, Debre Birhan Referral Hospital, and Boru Media District Hospital) in Amhara Region, North Ethiopia, from March 01-April 30, 2019. Amhara National Regional State is the second-largest region which is located in the Western and Northern Central parts of Ethiopia; it has 12 zones, three-city administrations, and 180 woredas (139 rural and 41 urban). According to the 2017 Ethiopian Central Statistics Agency report, the region has a projected population of 21.5 million people, about 80 percent of whom are rural farmers (23). The region has 80 hospitals (5 referral, 2 general, and 73 primary), 847 health centers and 3,342 health posts (23). Currently, a total of 14 MDR-TB treatments initiating centers are available, of which 3 randomly selected MDR-TB treatment initiating centers; namely, Debre Markos Referral Hospital, Debre Birhan Referral Hospital, and Boru Media District Hospital were included in this study. the study population for cases were All TB patients with culture or Drug Susceptibility Testing (DST) confirmed TB strains resistant to at least Isoniazid and Rifampicin registered on second-line TB treatment in the selected MDR-TB treatment initiating centers from January 01/2014 - December 31/2018. All TB patients with bacteriologically proven drug- susceptible TB strains, whose recent smears result were turned to negative at 6^th^ month and either 2nd or 5th months registered as cured from January 01/2014 up to December 31/2018 were the study population for controls.

For cases All confirmed MDR-TB patients, whose ages ≥18 years and registered on second- line anti-TB treatment from January 01/2014 upto December 31/2018 were included in the study. for controls All smear-positive TB patients, whose ages ≥18 years and complete their treatment in the same institution with cases and registered as cured from January 01/2014 to December 31/2018 were included.

Double population proportion formula was used to determine the sample size using Epi-Info version 7. HIV seropositivity was taken as the main research hypothesis variable and the sample was determined by considering the following assumptions: The critical value of 95% CI (Z= 1.96), power (Z1-β) = 80%, the ratio of case to control 1:3, percent of exposure among cases (P1 = 50%) and percent of exposure among controls (P2= 28.9%) (24). Based on the above assumptions, the largest calculated sample size for variable HIV seropositive was 244. Considering the 1.5 design effect and 10% non-response rate, the final sample size became 400 (100 cases and 300 controls).

The study participants were selected using multi-stage sampling technique at three randomly selected MDR-TB treatment initiating hospitals in the Amhara Region, Northern Ethiopia. The number of controls and cases from each MDR-TB treatment initiating center was allocated proportionally. After reviewing five years of TB patients' documents, a total of 208 cases and 321 controls fulfilled the inclusion criteria. After stratification of the population based on their disease's status, three controls were selected for each case. Generally, 100 cases and 300 controls were selected through simple random sampling technique using computer-generated random numbers. The outcome variable of this study was the presence/absence of Multi-Drug Resistant TB. The explanatory variables were age, sex, place of residence, previous history of TB treatment, degree of smear positivity, undernutrition, HIV status, Diabetes mellitus (DM) conditions and cigarette smoking status of the study participants.

Data were collected by two trained nurses from TB registration book and patient charts at a separate room of TB wards. The data extraction instrument was pretested on 5% of the total sample size. Data collections using the checklists were taken place at Debre Markose Referral Hospital before the actual data collection to check the validity and consistency of the checklist. Then, the checklist was modified based on the availabilities of registered data both at the TB registration book and patients' charts. The data collector was monitored by one supervisor and principal investigator during the data collection process.

The data were coded, entered and cleaned using Epi-Info version 7 and exported to Statistical Package for Social Science (SPSS) version 23. Data completeness and consistency were checked by running frequencies of each variable. In univariate analysis, cross-tabulation was also performed to see the distribution of each independent variable with the outcome variable. Simple binary logistic regression analysis was used to select candidate variables for multivariable logistic regression analysis. Variables with p-values of less than 0.25 were entered into the multivariable logistic regression analysis model to control the effect of different confounding factors while assessing the effect of each independent variable with the outcome variable. Adjusted Odd Ratio (AOR) along with 95% CI was used to determine the strength of association with the level of statistical significance p-value <0.05. The model goodness of fit was checked by Hosmer and Lemeshow test (P-value = 0.624). It was used with a stepwise backward likelihood ratio for multivariable logistic regression analysis. Ethical clearance was obtained from the school of Nursing Ethical Review Committee on behalf of the University of Gondar. A detailed explanation of the purpose, benefit, and risk of the research were given to the selected health facilities. Then, verbal consent was obtained from each MDR-TB treatment initiating center managers. Patient identification data were removed to maintain the study Participants' confidentiality.

## Results

**A descriptive study of the risk factors of MDR-TB**: Among the total calculated sample size, 393 (98.3%) (98 cases and 295 controls) were included in this study. The rest seven reviewed document were excluded from the study due to incomplete data.

The mean (+/-standard deviation [SD]) age of the total study antiparticles was 32.0 (+/-10.9) years, and males were found to be predominant, 216 (55%). The numbers of males among controls, 162(54.9%), and cases, 54(55.1%), were nearly equal. Twenty-three (23.5%), 69(70.4%) and 6(6.1%) of the cases were found in the age group of between 18–25, 26–45 and ≥46 years old respectively. The majority of cases 69(70.4%) were residing in rural than urban area 29 (29.6%). Similarly, in controls, more than half of the study participants, 164(55.6%), were living in rural than urban areas.

As shown in Table 1, 42(43.9%) cases and 120(40.7%) controls were found to be ≥+3 bacterial loads on their sputum per field. Furthermore, 75(76.5%) of the cases and 56(19%) of the controls had the previous history of TB treatment, and 22(22.4%) of the cases and 7(5.8%) of the controls were co-infected with HIV. According to BMI classification, 9(9.2%) cases and 20(6.8%) controls were under nutrished (BMI≤18.49). A total of nine (9.2%) cases and twenty (6.8%) controls were contracted with DM.

**Table 1:** Socio-demographic and medical characteristics of the study participants at selected MDR-TB initiating centers of Amhara region, Northern Ethiopia, January 01/2014 – December 31/2018, (N= 393)

Variables	MDR - TB cases, (N=98); N (%)	Non MDR - TB (controls) (N=295); N (%)
**Sex**		
Male	54 (55.1%)	162 (54.9%)
Female	44 (44.9%)	133 (45.1%)
**Age in years**		
18 – 25	23(23.5%)	89 (30.1 %)
26 – 45	69 (70.4%)	163(55.3%)
>46	6(6.1%)	43(14.6%)
**Place of Residence**		
Urban	29 (29.6%)	131 (44.4%)
Rural	69 (70.4%)	164 (55.6%)
**Degree of smear positivity**		
+2	56 (57.1%)	175 (59.3%)
+3	42 (43.9%)	120 (40.7%)
**Previous treatment history**		
Yes	75 (76.5%)	56 (19%)
No	23 (23.5%)	239 (81 %)
**HIV status**		
Positive	22 (22.4%)	17 (5.8%)
Negative	76 (77.6%)	278 (94.2%)
**BMI In kg/m2**		
≤18.49	9(9.2%)	32 (10.8%)
≥18.5	89 (90.8%)	263 (89.2%)
**Contracted with DM**		
Yes	9 (9.3%)	20 (6.8%)
No	89 (90. 8%)	2 75 (93.2%)
**smoking History**		
Yes	10(10.2%)	18 (6.1 %)
No	88 (89.8%)	277 (93.9%)

NB: BMI= Body mass index, kg/m^2^ = kilogram per meter square

**Bivariable and Multivariable analysis of the determinants of MDR-TB**: The result from the multivariable analysis showed that age 26–45 years old, previous history of TB treatment and HIV infection were significantly associated with MDR-TB in the cases as compared to the controls (Table 2).

**Table 2:** Bivariable and multivariable analysis of the determinants of MDR-TB at selected MDR- TB treatment initiating centers, Amhara Region, Northern Ethiopia (N=393)

Variables	MDR	Non	COR(95%CI)	AOR(95%CI)	P-value
	TB	MDR			
	(N)	TB (N)			
**Sex**					
Male	54	162	1.01 (0.64, 1.59)	1.14(0.78,2.52)	0.25
Female	44	133	1	1	
**Age in years**					
18–25	23	89	1.85(0.70,4.88)	2.39(0.76,7.58)	0.14
26–45	69	163	3.03(1.23,7.46)	3.35(1.15,9.78)	0.03*
≥46	6	43	1	1	
**Place of** **residence**					
Urban	29	131	0.21(0.32, 0.86)	0.62(0.34,1.12)	0.12
Rural	69	164	1	1	
**Degree of** **smear positive**					
≤+2	56	175	1	1	
≥+3	42	120	0.91(0.69, 1.74)	1.51(0.84,2.71)	0.17
**Previous TB** **treatment**					
Yes	75	56	13.9(8.03,24.13)	14.16(7.8,25.4)	0.001*
No	23	239	1	1	
**HIV status**					
Positive	22	17	4.73 (2.39,9.36)	4.39(1.85,10.4)	0.001*
Negative	76	278	1	1	
**Body mass index**					
≤18.49	9	32	0.83(0.38, 1.80)	0.83(0.30, 2.26	0.52
≥18.5	89	263	1	1	1.000
**Diabtes Millitues**					
Yes	9	20	1.39 (0.61,3.16)	1.33(0.48,3.74)	0.58
No	89	275	1	1	
**Smoking history**					
Yes	10	18	1.75(0.78, 3.93)	2.29(0.79,6.69)	0.13
No	88	277	1	1	

-N = number of the study subjects, COR = crude odd ratio, AOR = Adjusted odd ratio

* indicates statistically significant at multivariate analysis (p<0.05)

People whose ages were between 26–45 years old were three times more likely to develop MDR-TB (AOR=3.3; 95% CI: 1.15, 9.77) compared to people whose ages were above 46 years. Those people who had a previous history of TB treatment were also fourteen times more likely to develop MDR-TB than those who have not been treated before (AOR=14.16; 95%CI:7.80,25.38). MDR-TB was significantly associated with HIV infection. People who were co-infected with HIV were 4.4 times more likely to develop MDR-TB than those TB patients who were HIV negatives [AOR=4.38; 95%CI: (1.85,10.38)].

## Discussion

The determinants of MDR-TB can vary according to the type of study design and the countries where the studies were conducted (12,15). Besides that, although Ethiopia is one of the 30 MDR-TB high burden countries, there were limited studies with soundable study design and larger samples to identify the risk factors of this disease in the local context (22). In the current study, age between 26–45 years old, previous history of TB treatment and coinfection with HIV were identified as the risk factors for MDR-TB. The study revealed that those people whose ages were between 26–45 years old were 3.4 times more likely to develop MDR-TB compared to age older than 45 years (AOR=3.35; 95% CI: 1.15, 9.77). This finding is in line with a study done in Eastern Ethiopia (AOR =2.74) (25). The positive association of this age group with MDR-TB could be due to the fact that this age group has a higher tendency not to adhere to anti-TB medication because of tightness with work, travel of long distance to search work and addiction to alcohol compared to older people whose lifestyle is sedentary.

In contrast to this finding, a single study in Ethiopia showed that people whose ages were between 18–44 years old had no difference in developing MDR-TB between cases and controls (26). This discrepancy might be related to the difference in study design and age classification.

On the other hand, findings from Spain (27) and China (28) revealed that those people whose ages were older than 45 years old appears to be the predisposing factor for MDR-TB compared to age between 26–45 years old. This is explained by a difference in population distribution and the health policy of the country.

The finding in this study showed that those TB patients who had previous history TB treatment were fourteen times at higher risk of developing MDR-TB than those who had never been treated for TB in their life (AOR= 14.16; 95%CI: 7.80,25.38). This finding is comparable with data reported from Addis Ababa, Ethiopia (21,25) and Jimma in Ethiopia (24). The possible reason could be poor treatment adherence and drug storage as also evidenced for the possible cause of emerging MDR-TB (13).

This finding is also in line with studies conducted from Namibia (29), Bangladesh (30), Georgia (31), China (32) and Malaysia (33). The possible justification could be repeated and inappropriate way of taking anti-TB medication that mutates the structure of the bacterial gene and develops resistance against the drugs (16,24).

In this study, we also found that being HIV positive increases the risk of developing MDR-TB by 4.4 times compared to HIV negative TB patients. The finding is consistent with studies conducted in Eastern Ethiopia (AOR= 2.32) (34)nd A addis Ababa, Ethiopia (AOR=3.1) (25). The possible reason could be that those TB patients co-infected with HIV might have high risk of exposure to MDR-TB cases during hospitalization and frequent health institution visiting (35). There is also high burden of TB in HIV positive than in HIV negative in our settings (5). This finding is also consistent with a study done in Netherland (36). The association between co-infection with HIV and MDR-TB could be due to alteration of the immune system that leads to an increase in the chance of developing MDR-TB and anti-TB drugs malabsorption (35).

Contrary to the current finding, some studies revealed that being HIV positive was not significantly associated with the occurrence of MDR-TB (17,20,31). The discrepancy of this finding could be inadequate testing of MDR-TB (14). There is also an alternative possible justification for this discrepancy that might be a difference in the study design and socio-economical characteristics of the countries. Likewise, co-nfection with HIV infection had no association with the occurrence of MDR-TB according to a systematic and meta-analysis study done on 32 studies in South Africa (19). The possible justification might be that the Meta-analysis was done among studies with a cross-sectional study design which is a poor design for studying associations (cause-effect relationship), unlike a case-control study which we have used in our study and relatively good for studying determinants than cross-sectional study.

Generally, in this study, age between 26–45 years old, previous history of TB treatment and co-infection with HIV were identified as the determinants for MDR-TB. Therefore, special attention should be given for younger age groups of the population, previous history of TB treatment and co-infected with HIV to prevent and control national MDR-TB burden according to the local context to keep the community healthy and to avert the rising problem in Amhara Region as well as the country.

Inability to include some important variables in this study (like frequency of hospitalization, treatment adherence, and alcohol consumption) because of the incompleteness of data about these variables on registration book and patient charts was the limitation of this study.
